# Sitagliptin, An Anti-diabetic Drug, Suppresses Estrogen Deficiency-Induced Osteoporosis*In Vivo* and Inhibits RANKL-Induced Osteoclast Formation and Bone Resorption *In Vitro*

**DOI:** 10.3389/fphar.2017.00407

**Published:** 2017-06-30

**Authors:** Chuandong Wang, Fei Xiao, Xinhua Qu, Zanjing Zhai, Guoli Hu, Xiaodong Chen, Xiaoling Zhang

**Affiliations:** ^1^Department of Orthopedic Surgery, Xin Hua Hospital Affilliated to Shanghai Jiao Tong University School of Medicine (SJTUSM)Shanghai, China; ^2^Shanghai Key Laboratory of Orthopedic Implant, Department of Orthopedic Surgery, Shanghai Ninth People’s Hospital, Shanghai Jiao Tong University School of Medicine (SJTUSM)Shanghai, China; ^3^The Key Laboratory of Stem Cell Biology, Institute of Health Sciences, Shanghai Jiao Tong University School of Medicine (SJTUSM) and Shanghai Institutes for Biological Sciences (SIBS), Chinese Academy of Sciences (CAS)Shanghai, China

**Keywords:** sitagliptin, osteoclast, osteoporosis, NFATc1, RANKL

## Abstract

Postmenopausal osteoporosis is a disease characterized by excessive osteoclastic bone resorption. Some anti-diabetic drugs were demonstrated for anti-osteoclastic bone-loss effects. The present study investigated the skeletal effects of chronic administration of sitagliptin, a dipeptidyl peptidase IV (DPP IV) inhibitor that is increasingly used for type 2 diabetes treatments, in an estrogen deficiency-induced osteoporosis and elucidated the associated mechanisms. This study indicated that sitagliptin effectively prevented ovariectomy-induced bone loss and reduced osteoclast numbers *in vivo*. It was also indicated that sitagliptin suppressed receptor activator of nuclear factor-κB ligand (RANKL)-mediated osteoclast differentiation, bone resorption, and F-actin ring formation in a manner of dose-dependence. In addition, sitagliptin significantly reduced the expression of osteoclast-specific markers in mouse bone-marrow-derived macrophages, including calcitonin receptor (*Calcr*), dendrite cell-specific transmembrane protein (*Dc-stamp*), *c-Fos*, and nuclear factor of activated T-cells cytoplasmic 1 (*Nfatc1*). Further study indicated that sitagliptin inhibited osteoclastogenesis by suppressing AKT and ERK signaling pathways, scavenging ROS activity, and suppressing the Ca^2+^ oscillation that consequently affects the expression and/or activity of the osteoclast-specific transcription factors, *c-Fos* and NFATc1. Collectively, these findings suggest that sitagliptin possesses beneficial effects on bone and the suppression of osteoclast number implies that the effect is exerted directly on osteoclastogenesis.

## Introduction

Bone is dynamically remodeled throughout life by the complementary activities of bone-forming osteoblasts and bone-resorbing osteoclasts. The imbalance between osteoblast and osteoclast formation and function during skeletal turnover leads to various osteopathic diseases, particularly bone loss caused by excessive bone resorption, such as osteoporosis and Paget’s disease (Feng and McDonald, 2011). Osteoclasts are the main type of bone-resorbing multinucleated giant cells derived from hematopoietic progenitors upon stimulation by two essential cytokines: the RANKL and the M-CSF (Kong et al., 1999; Boyle et al., 2003). M-CSF is critical in providing signals of survival and proliferation to osteoclast precursor cells and promoting the expression of RANK, which is a prerequisite for osteoclast differentiation. The binding of RANK and RANKL triggers RANK trimerization and subsequently activates the tumor necrosis factor receptor associated factor 6 (TRAF6); these events activate multiple downstream signaling pathways, such as NF-κB, AKT, and mitogen-activated protein kinases (MAPKs), including the JNK, p38, and ERK; ultimately, this cascade activates the transcription factors *c-Fos* and NFATc1, which are required for osteoclast differentiation (Boyle et al., 2003; Zaidi et al., 2003). The RANKL–RANK axis also stimulates the Ca^2+^–NFATc1 signaling pathway by activating PLCγ. During osteoclastogenesis, activated PLCγ hydrolyzes phosphatidylinositol-4,5-biphosphate (PIP2) to inositol-1,4,5-triphosphate (IP3), thereby upregulating both the intracellular Ca^2+^ and the NFATc1 activation (Negishi-Koga and Takayanagi, 2009; Hwang and Putney, 2011; Kim et al., 2014, 2015). Oxidative stress stimuli transmitted by increased intracellular production of ROS could play as the secondary messengers in RANKL-induced osteoclast signaling pathways (Lee et al., 2005). By regulating these early signaling cascades, NFATc1 induces the expression of various osteoclast-specific genes, such as *Dc-stamp*, *Calcr*, *Nfatc1* and *c-Fos* (Zhai et al., 2014; Zhou et al., 2016).

As an oral anti-diabetic agent, sitagliptin is commonly used as mono-therapy or in combination therapy with other oral hypoglycemic agents in the treatment of T2DM by inhibiting DPP IV activity (Barnett, 2006). The pharmacological effect of DPP-4 inhibitors is to prolong the action of incretin hormones, which increase insulin synthesis and release from pancreatic β cells (Meier et al., 2016). Increasing blood incretin levels may have direct beneficial effects on bone (Kyle et al., 2011). It was recently reported that sitagliptin would reduce bone loss and increase bone strength in diabetic rats by reducing bone resorption independent of glycemic management (Glorie et al., 2014; Baerts et al., 2015). Consistent with animal studies, human studies indicate that sitagliptin could positively regulate bone metabolism and reduce the fracture risk (Monami et al., 2011; Hegazy, 2015). However, the underlying mechanisms of the bone protection effects by sitaglipin remain unclear.

Given the inhibitory effect of sitagliptin on diabetic osteoporosis bone loss and the important role of osteoclasts in bone homeostasis regulation, we hypothesized that sitagliptin could regulate bone homeostasis by inhibiting osteoclast differentiation. In this study, we provided evidence that sitagliptin suppresses the estrogen deficiency-induced osteoporosis by reducing osteoclastogenesis in the OVX mouse model; we also elucidated its cellular and molecular mechanisms of action *in vitro*.

## Materials and Methods

### Animals and Study Design

Animal studies were conducted according to procedures and principles approved by the Animal Care Committee of Shanghai Jiao Tong University, Shanghai, China. A total of 20 specific pathogen-free female C57BL/6J mice, with the age of 12 weeks, were included in this study and were randomly divided into four groups: the OVX group, the OVX+sitagliptin (10 mg/kg body weight of each) group, the OVX+sitagliptin (25 mg/kg body weight of each) group and the sham group. Each group contained five mice. A sham operation was conducted in the sham group as a control, whereas those in the other three groups underwent an ovariectomy (Zhou et al., 2016). The animals were maintained under standard animal housing conditions (12 h light/dark cycles and free access to food and water). Four weeks later, the mice in the OVX+sitagliptin groups received daily intragastric administration of sitagliptin dissolved in saline at the indicated concentration, whereas the mice in the sham and OVX groups were treated with saline as a control. After four weeks, all the mice were euthanized and their tibias were collected for further analysis. Right tibias were dissected for micro-CT analysis and left tibias for bone histomorphometry. The success of ovariectomy was confirmed by observing uterine atrophy during dissection.

### Micro-CT Analysis of Tibias

Right tibias were fixed in 4% paraformaldehyde in PBS (pH 7.4) for 24 h and stored in 70% ethanol at 4°C. The samples were scanned by high-resolution micro-computed tomography (μCT) (SCANCO μCT 100, Brüttisellen, Switzerland). Each tibia was imaged with the following instrument settings: 70 kV, 200 μA, 0.5 mm Al filter, 300 ms exposure, and 5 μm pixel size. After scanning, the data were reconstructed with SCANCO μ100 Evaluation V6.5-3 with a constant threshold value. A volume of interest was generated 0.5 mm in height and 0.25 mm below the growth plate. The trabecular bone region of interest (ROI) within this volume was manually defined and the following bone parameters were included: Tb.Th, BMD, bone volume percentage (BV/TV), Tb.N, SMI, and Tb.Sp.

### Histological and Histomorphometric Analysis

Left tibias were fixed in 4% paraformaldehyde in PBS (pH 7.4) for 24 h, and then 10% EDTA was used for decalcification for approximately 1 month. The tissues were embedded in paraffin and histological sections (7 μm thick) were made. Subsequently the sections underwent hematoxylin and eosin (H&E) and TRAP staining with hematoxylin counterstain. A high-quality microscope was used to examine and photograph the specimens. The number of TRAP^+^ multi-nucleated osteoclasts (N.Oc/BS, 1/mm) and the percentage of osteoclast surface per bone surface (OcS/BS, %) were assessed for each sample.

### Media, Reagents, and Antibodies

Sitagliptin was purchased from Meilun Biotech (Dalian, China) and dissolved in normal saline. Soluble mouse recombinant M-CSF and RANKL were obtained from R&D Systems (Minneapolis, MN, United States). Fetal bovine serum (FBS), alpha-MEM and penicillin were purchased from Gibco BRL (Gaithersburg, MD, United States). Triton X-100, 4′,6-diamidine-2′-phenylindole dihydrochloride (DAPI) and TRAP staining solution were obtained from Sigma–Aldrich (St. Louis, MO, United States). Cell Counting Kit-8 (CCK-8) was purchased from Dojindo Molecular Technology (Kumamoto, Japan). The Acti-stain 555 fluorescent phalloidin was obtained from Cytoskeleton Inc. (Denver, CO, United States). The Cell Tracker Green, Fluo-4 AM, Hank’s balanced salt solution (HBSS), membrane dye DiI, and Pluronic F-127 were obtained from Life Technologies (Carlsbad, CA, United States). Primary antibodies and secondary antibodies were obtained from Cell Signaling Technology (Danvers, MA, United States). Dichlorofluorescin diacetate (DCFDA) cellular ROS detection assay was purchased from Beyotime (Shanghai, China).

### Survival and Proliferation Assay

Bone marrow macrophages (BMMs) were prepared according to the previous study (Xiao et al., 2015). Under the manufacturer’s instructions of the CCK-8 kit, the assessment of the anti-proliferative effect of sitagliptin on BMMs was accomplished. Briefly, BMMs were seeded in triplicate in 96-well plates at a density of 1 × 10^4^cells/well and then cultured in 100 μL of complete α-MEM medium for 24 h or 72 h, with different doses of sitagliptin (0, 1.56, 3.12, 6.25, 12.5, 25, or 50 μg/mL). Subsequently, each well was added with 90 μL FBS-free α-MEM medium mixed with 10 μL CCK-8 reagent. Two hours’ incubation after that, each well was sent for analysis of optical density (OD) at 450 nm (630 nm as reference) with an ELX800 absorbance microplate reader (Bio-Tek, Winooski, VT, United States).

### *In Vitro* Osteoclastogenesis Assay

BMMs were differentiated over 5 d into osteoclasts by incubation in complete culture medium containing 10% FBS, 100 U/mL penicillin, RANKL (50 ng/mL), and M-CSF (30 ng/mL), as previously described (Xiao et al., 2015). BMMs were plated in 96-well plates at a density of 8 × 10^3^ cells per well and allowed to adhere overnight. To determine the influence of sitagliptin on osteoclast differentiation, the drug was included in the culture medium at a range of concentrations (0, 12.5, 25, or 50 μg/mL). Every second day of incubation, the culture medium was replenished with fresh medium containing RANKL, M-CSF, and the indicated concentration of sitagliptin. To investigate any stage of sitagliptin addition-dependent effects on osteoclastogenesis, BMM cells were cultured with sitagliptin (50 μg/mL) added at different stages of the 5 day culture period: 12 h before RANKL (pre-treatment), day 1 (early treatment), day 3 (late treatment), days 1 and 3 (early + late treatment) and 12 h before RANKL treatment, days 1 and 3 (pre + early + late treatment). After 5 days, cells were fixed in paraformaldehyde and stained for TRAP activity. The number of mature osteoclasts (TRAP^+^ cells containing more than three nuclei) was calculated and the spread area (mm^2^) was also measured.

### Reverse Transcription-Quantitative Polymerase Chain Reaction (RT-qPCR)

BMMs with a density of 1 × 10^5^ were plated in each well of a 24-well plate. The complete culture medium contained 10% FBS, α-MEM, M-CSF (30 ng/mL), RANKL (50 ng/mL), and 100 U/mL penicillin. Then the cells were treated with sitagliptin at a range of concentrations (0, 12.5, 25, or 50 μg/mL). Total RNA was extracted by using an RNeasy Mini kit (Qiagen, Valencia, CA, United States). One microgram of total RNA was used to synthesize cDNA by reverse transcription (TaKaRa Biotechnology, Otsu, Japan). SYBRPremix Ex Tag kit (TaKaRa Biotechnology) and ViiA 7Real-time PCR machine (Applied Biosystems, Warrington, Uniked Kingdom) were used for qPCR. PCR was performed under the following conditions: 40 cycles with 5 s of denaturation at 95°C and 34 s of amplification at 60°C. The expression levels were normalized to *Gapdh*. The following primer sets were used as previously described (Zhai et al., 2014): mouse Calcr: forward, 5′-TGGTTGAGGTTGTGCCCA-3′, and reverse, 5′-CTCGTGGGTTTGCCTCATC-3′; mouse DC-STAMP: forward, 5′-AAAACCCTTGGGCTGTTCTT-3′, and reverse, 5′-AATCATGGACGACTCCTTGG-3′; mouse NFATc1: forward, 5′-CCGTTGCTTCCAGAAAATAACA-3′, and reverse, 5′-TGTGGGATGTGAACTCGGAA-3′; mouse *c-Fos*: forward, 5′-CCAGTCAAGAGCATCAGCAA-3′, and reverse, 5′-AAGTAGTGCAGCCCGGAGTA-3′; mouse GAPDH: forward, 5′-ACCCAGAAGACTGTGGATGG-3′, and reverse, 5′-CACATTGGGGGTAGGAACAC-3′.

### Analysis of Apoptosis by Flow Cytometry and DAPI Staining

Cell apoptosis was assessed with Annexin V/propidium iodide (PI) (Invitrogen Life Technologies, United States). BMMs were exposed to sitagliptin (0, 12.5, 25, or 50 μg/mL) for 24 and 48 h, respectively, and then cells were suspended in a binding buffer. Five microliter of annexin V-FITC and 10 μL of 20 μg/mL PI were used to stain the treated BMMs for 15 min, which was processed at room temperature (RT) in the dark place. Cells were excited at 488 nm, and signals from 10,000 cells were obtained. Results were analyzed with FACStar (BD Biosciences). BMMs were treated with sitagliptin (0, 12.5, 25, or 50 μg/mL) for 48 h. PBS was used to rinse the cells for three times. Triton X-100 was used to destroy the cell membrane integrity for 15 min. Nuclear stained with 0.1 μg/mL DAPI (Beyotime, Shanghai, China) in PBS at 37°C for 10 min in the dark. The cell nuclei were observed and photographed with a LSM5 confocal microscope (Carl Zeiss, Oberkochen, Germany).

### Resorption Pit Assay and F-Actin Ring Formation Assay

Equal numbers of BMMs-derived pre-osteoclasts, which were stimulated with RANKL for 4 d, were seeded onto devitalized bovine bone disks and allowed to adhere overnight. Cultures were then treated with sitagliptin (0, 12.5, 25, or 50 μg/mL) for 48 h. Cells that had adhered to bone slices were removed by mechanical agitation and sonication. Resorption pits visualized under a scanning electron microscope (FEI Quanta 250) and the bone resorption area was quantified with Image J software (NIH, Bethesda, MD, United State) and expressed as a percentage of the total area of bone disk. To visualize F-actin rings, equal numbers of BMM-derived pre-osteoclasts (4 days RANKL stimulation) were seeded onto a covered glass-bottomed dish. Osteoclasts were fixed in 4% paraformaldehyde for 15 min after 24–48 h’s treatment with sitagliptin (0, 12.5, 25, or 50 μg/mL), and then permeabilized with 0.1% Triton X-100 for 5 min. After that, incubation of the cells was done in rhodamine-conjugated phalloidin for 15 min (Xiao et al., 2015), followed by incubation with DAPI dye (1:10000; Invitrogen Life Technologies) and PBS wash. Then cells were mounted with ProLong Gold anti-fade mounting medium (Invitrogen Life Technologies). LSM5 confocal microscope (Carl Zeiss, Oberkochen, Germany) was used to visualize the actin ring distribution.

### Intracellular ROS Detection

DCFDA cellular ROS detection assay kit (Beyotime, Shanghai, China) was used to detect the intracellular ROS levels following the manufacturer’s instructions. The BMMs (8 × 10^3^ cells/well in 96-well plates) of treatment groups were treated with RANKL, M-CSF, and sitagliptin (25 or 50 μg/mL). The positive control group cells were treated with M-CSF and RANKL, whereas the vehicle group cells were only treated with M-CSF. After incubation for 72 h, the cells in each group were washed with PBS, followed by incubation in dark with 10 μM DCFH-DA for 1 h. Intracellular ROS levels were detected with 2’,7’-dichlorofluorescein diacetate (DCFH), which would be oxidized into fluorescent DCF with the presence of ROS. Images were obtained with a fluorescence microscope (Carl Zeiss, Oberkochen, Germany).

### Intracellular Ca^2+^ Oscillation Measurement

Intracellular Ca^2+^ oscillation measurement was performed in accordance with a previous study (Dou et al., 2016). Briefly, BMMs were plated on 96-well covered glass-bottomed plates with a density of 8 × 10^3^ cells, treated with supplements based on the assigned groups. Cells in the treatment groups were treated with M-CSF, RANKL, and sitagliptin (50 μg/mL); positive control group cells were treated with M-CSF and RANKL, whereas cells in the vehicle group were only treated with M-CSF. After 3 days, the cells were incubated in Fluo-4 AM (5 μM) and 0.05% Pluronic F-127 (Invitrogen) in HBSS added with 1% FCS/1 mM probenecid (assay buffer) for 30 min at 37°C. Cells were washed by the assay buffer for three times and underwent RT for 15 min. An inverted fluorescent microscope (Olympus) was used for exciting samples at 488 nm. The relative intracellular calcium level was monitored for 3 min with 5 s’ interval based on the fluorescence intensity of Fluo-4 at 200× magnification. Oscillating cells were those that performed at least two oscillations. At least 40 cells were monitored in each well for three times. The average amplitude of calcium oscillation in each cell was calculated with the TuneR and SeeWave packages for the R programming language (Dolmetsch and Lewis, 1994).

### Western Blot Analysis

Western blot analysis was performed as previously described (Xiao et al., 2015). RAW264.7 cells were seeded in 6-well plates at a density of 6 × 10^5^ cells per well. After growth to confluence, the cells were pretreated with or without sitagliptin for 4 h. Cells were then stimulated with 50 ng/mL RANKL for 0, 5, 10, 20, 30, or 60 min. Cells were lysed with radioimmunoprecipitation assay lysis buffer (ThermoFisher Scientific, Waltham, MA, United States), and the protein concentration was determined with a BCA protein assay (Thermo FisherScientific). Lysate proteins (30 μg) were separated by 10% SDS-PAGE and transferred to polyvinylidene difluoride membranes. The membranes were blocked with 5% skimmed milk in TBST (TBS: 0.05 M Tris; 0.15 M NaCl, pH 7.4; 0.1% Tween-20) for 1 h, and incubated with primary antibodies diluted in 5% (w/v) skimmed milk powder in TBST overnight at 4°C. HRP-conjugated secondary antibodies were used at a 1:5000 dilution. The antigen–antibody complexes were visualized with the enhanced chemiluminescence detection system (Millipore, Billerica, MA, United States). Immunoreactive bands were quantified on scanned films in triplicate with the help of Alpha Image software by normalizing the band intensities to GAPDH.

### Statistical Analysis

SPSS 19.0 software (SPSS Inc., United States) was included for statistical analysis. All values are presented as the mean ± standard deviation (SD) from three or more independent replicates. Student’s *t*-test was performed to compare the difference. ^∗^*p* < 0.05 and ^∗∗^*p* < 0.01 indicated significantly difference.

## Results

### Effects of Sitagliptin on OVX-Induced Bone Loss

**FIGURE 1:**
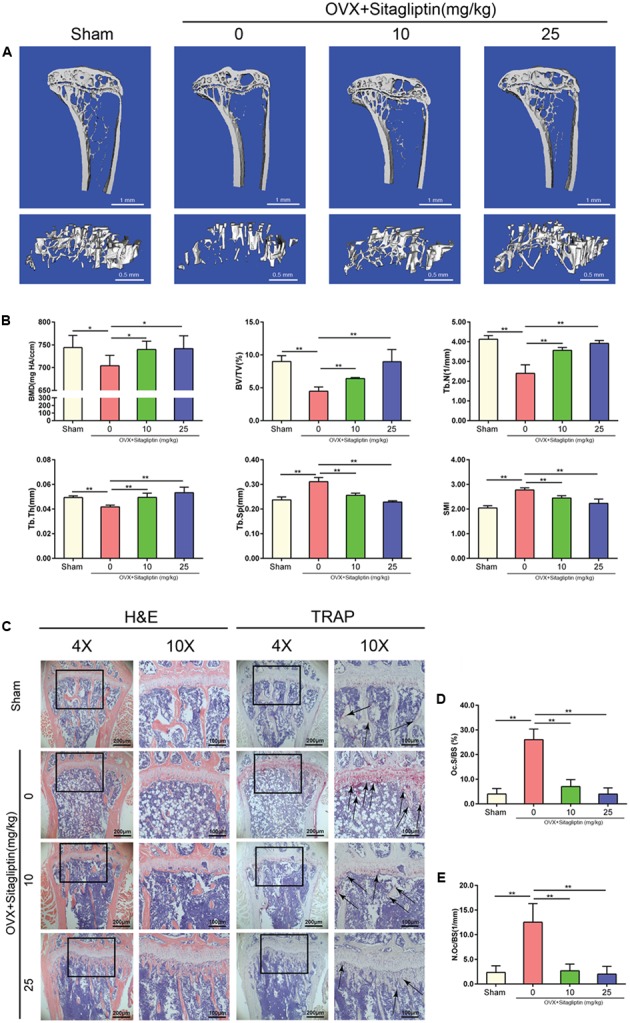
Sitagliptin protects against ovariectomy-induced bone loss by inhibiting osteoclast activity. **(A)** Frontal sections through the proximal tibial metaphyses (top panel, 0.25 mm thick; scale bar: 1 mm) and 3D reconstructions of trabecular bone (lower panel, scale bar: 500 μm) from the tibia of sham mice, OVX mice, and OVX mice treated with 10 mg/kg and 25 mg/kg sitagliptin. **(B)** Quantitative analyses of BMD, bone volume/total volume, Tb.N, Tb.Th, Tb.Sp, and SMI, *n* = 5. **(C)** Representative images of decalcified bone stained with H&E and TRAP (black arrowheads) from sham mice, OVX mice, and OVX mice treated with 10 mg/kg and 25 mg/kg sitagliptin. Magnification = 4×, scale bar = 200 μm; magnification = 10×, scale bar = 100 μm. **(D)** Quantitative analyses of osteoclast surface/bone surface (Oc.S/BS), *n* = 5. **(E)** Quantitative analyses of osteoclast number/bone surface, *n* = 5. Data are expressed as mean ± SD; ^∗∗^*p* < 0.01 relative to untreated controls.

To evaluate the role of sitagliptin in postmenopausal osteoporosis, we created the OVX-induced mouse bone-loss model. There were no fatalities after OVX and sitagliptin administration. All of the animals remained normal activity during the whole experiment. It was confirmed by Micro-CT that there was significant loss of tibia trabecular bone in OVX mice, which was indicated from the decreased BMD, BV/TV, Tb.Th, and Tb.N as well as the increased Tb.Sp as compared to sham-operated mice (**Figures 1A,B**). However, the extent of bone loss was significantly suppressed by treatment with sitagliptin in a manner of dose-dependence. Bone loss in mice treated with high sitagliptin concentration was markedly less than those treated with low sitagliptin concentration as compared with OVX mice treated with the vehicle (**Figures 1A,B**). In agreement with μCT analysis, the histological H&E-stained cells revealed that trabeculae in regions proximal and distal to the growth plate were scarce and thin in the OVX mouse group (**Figure 1C**). By contrast, OVX mice treated with sitagliptin presented a dramatic increase in bone density as well as a marked increase in trabecular density and thickness compared with OVX mice treated with the vehicle (**Figure 1C**).

To further explore whether osteoclasts were involved in the inhibitory effects of sitagliptin on OVX-induced bone loss, we performed TRAP staining on tibia bone sections. Accordingly, TRAP staining revealed the significant decrease of TRAP^+^ multinucleated cells at the growth plates and trabecular surface (**Figure 1C**) for sitagliptin-treated mice as compared with vehicle-treated OVX mice in a dose-dependent manner. Moreover, histomorphometric analysis of Oc.S/BS and the number of osteoclasts confirmed that sitagliptin treatment attenuated OVX-induced bone loss and reduced osteoclast numbers (**Figures 1C–E**). Collectively, these results indicated that sitagliptin effectively prevented OVX-induced bone loss *in vivo*.

### Effects of Sitagliptin on RANKL-Induced Osteoclastogenesis

**FIGURE 2:**
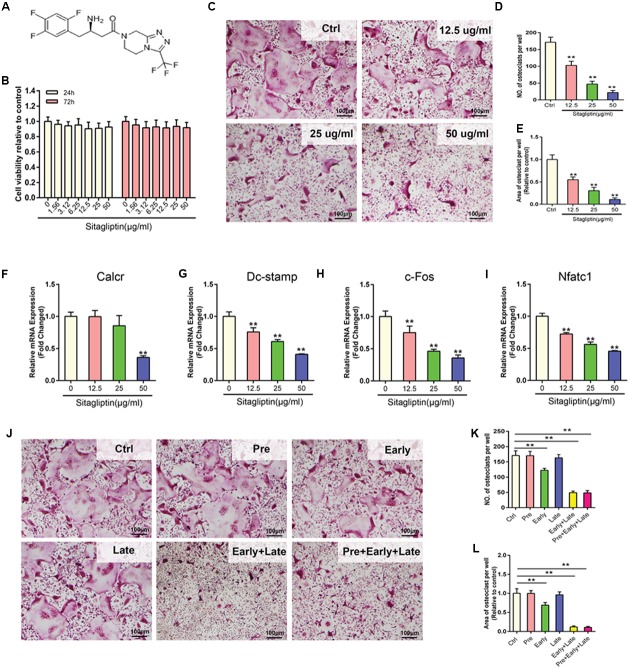
Sitagliptin inhibits RANKL-induced osteoclastogenesis *in vitro.* **(A)** Molecular structure of sitagliptin. **(B)** CCK-8 was performed in triplicate to analyze the cell viability of BMMs treated with varying doses of sitagliptin for 24 h and 72 h. **(C)** Representative images of BMMs stained for TRAP (red) and treated with sitagliptin at different doses (0, 12.5, 25, 50 μg/mL). Three independent experiments were performed. The number **(D)** and average cell spread area **(E)** of TRAP positive multinucleated osteoclasts (≥3 nuclei) were quantified. Sitagliptin suppresses the RANKL-induced expression of osteoclast specific genes; real-time PCR was used to determine the expression of osteoclast marker genes *Calcr* **(F)**, *Dc-stamp* **(G)**, *c-Fos* **(H)**, and *Nfatc1* **(I)**. The mRNA expression was normalized to GAPDH mRNA expression and converted to fold change of control. **(J)** Effect of time of adding sitagliptin on osteoclast formation. BMM cells were stimulated with RANKL (50 ng/mL) alone or co-treated with sitagliptin (50 μg/mL) at different stages of the 5-days osteoclast culture as described in methods. Cells were fixed and stained for TRAP activity. The number **(K)** and average cell spread area **(L)** of TRAP-positive multinucleated osteoclasts (≥3 nuclei) were quantified. All experiments were performed at least thrice and a representative image is shown. Data are expressed as mean ± SD; ^∗∗^*P* < 0.01 versus control. Scale bars: 100 μm.

To unveil the mechanisms of how sitagliptin prevents OVX-induced bone loss, the effect of sitagliptin on osteoclastogenesis was investigated *in vitro*. Concentrations of up to 50 μg/mL sitagliptin had no cytotoxic effects on BMMs, which were treated with sitagliptin for 24 and 72 h (**Figures 2A,B**). BMMs were treated with M-CSF and RANKL in the absence or presence of different concentrations of sitagliptin for 5 days. Numerous TRAP^+^ multinucleated osteoclasts were formed in the control group, whereas the osteoclast formation was inhibited by sitagliptin treatment in a dose-dependent manner (**Figures 2C–E**). Similarly, sitagliptin also dose-dependently suppressed the expression of osteoclast marker genes, including *Calcr*, *Dc-stamp*, *c-Fos*, and *Nfatc1* (**Figures 2F–I**).

To examine the stage at which sitagliptin inhibited osteoclastogenesis, cells were pretreated with 50 μg/mL sitagliptin for 12 h before RANKL stimulation; late treatment on the third day of RANKL stimulation was also conducted under the same condition. This study demonstrated that neither pre-treatment nor late treatment had effects on the size or number of TRAP^+^ osteoclasts after 5 days’ culture (**Figures 2J–L**). However, a significant decrease in the number and size of TRAP^+^ multinucleated osteoclasts was recorded on the first day of RANKL stimulation (early treatment) (**Figures 2J–L**). Additionally, consecutive exposure to sitagliptin for 2 days (early and late treatments) or 3 days (pre-, early, and late treatments) strongly suppressed the osteoclast formation (**Figures 2J–L**). Taken together, these results indicated that the inhibitory effects of sitagliptin are not long-term; effective suppression of osteoclast formation needs a maintained sitagliptin action during the whole process of RANKL-induced differentiation.

**FIGURE 3:**
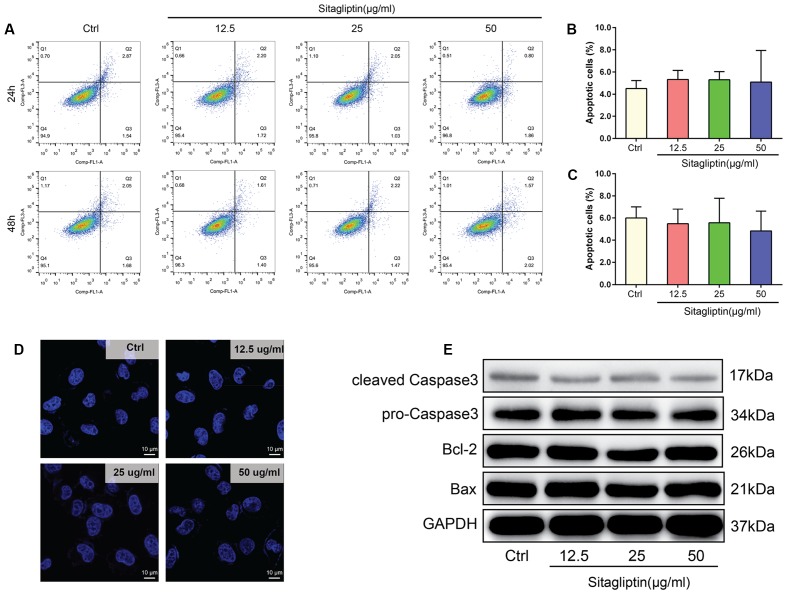
Sitagliptin does not induce apoptosis at concentrations that affect osteoclast formation and activity. **(A)** FCM analysis of the apoptosis rate of BMMs treated with varying doses of sitagliptin (0, 12.5, 25, and 50 μg/mL) for 24 and 48 h. Quantitative analysis of apoptosis rates of BMMs at **(B)** 24 h and **(C)** 48 h. **(D)** Sitagliptin did not induce nuclear fragmentation. BMMs cultured on glass cover slips were treated with various doses of sitagliptin (0, 12.5, 25, and 50 μg/mL) for 48 h before the cells were fixed, stained with 0.1 μg/mL DAPI, and examined by fluorescence microscopy. Scale bars: 10 μm. **(E)** Western blot analysis of cleaved caspase 3, pro-caspase 3, Bcl-2, and Bax protein expression in BMMs treated with various doses of sitagliptin (0, 12.5, 25, and 50 μg/mL) for 48 h. GAPDH was used as the loading control. Representative images of three repeated experiments are shown. Data are expressed as mean ± SD.

To exclude the possibility that sitagliptin induced BMM apoptosis during differentiation, BMMs were treated under a range of sitagliptin concentrations for 24 or 48 h. The number of Annexin-V^+^ cells was evaluated by flow cytometry. BMMs treated with a sitagliptin concentraion of 12.5–50 μg/mL showed a suppressed osteoclast formation, but the number of Annexin-V^+^ BMMs was not affected by sitagliptin treatment at either 24 or 48 h compared with the control group (**Figures 3A–C**). Furthermore, this study investigated whether sitagliptin treatment activate the apoptotic cell death pathways. There were no changes in the levels of the anti-apoptotic protein Bcl-2, and the Bax and caspase-3 apoptotic pathways were not activated after treatment with as much as 50 μg/mL sitagliptin (**Figure 3E**). BMMs stained with DAPI exhibited normal intact nuclei after sitagliptin treatment (**Figure 3D**), thereby confirming that the inhibitory effects of sitagliptin on osteoclastognesis were not caused by the induction of BMM apoptosis.

### Effects of Sitagliptin on Osteoclastic Bone Resorption

**FIGURE 4:**
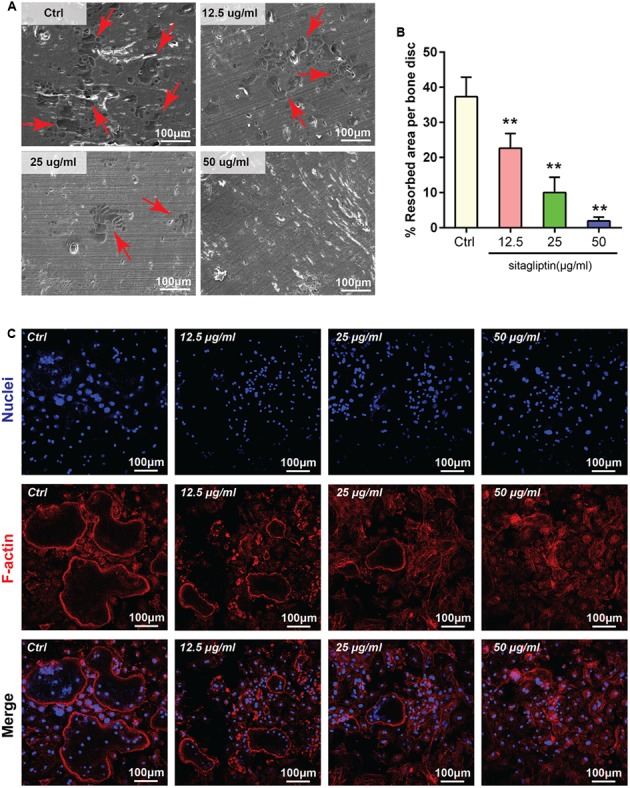
Sitagliptin attenuated osteoclastic bone resorption and F-actin ring formation *in vitro.* **(A)** Representative SEM images of bone resorption pits (red arrowheads) treated with varying doses of sitagliptin (0, 12.5, 25, and 50 μg/mL). Scale bars: 100 nm. **(B)** The total areas of the resorption pits were quantified. **(C)** BMMs were incubated with M-CSF (30 ng/mL) and RANKL (50 ng/mL) and then treated with varying doses of sitagliptin (0, 12.5, 25, and 50 μg/mL). Cells were fixed and stained with DAPI (nuclei) and rhodamine-phalloidin (F-actin). Scale bars: 100 μm. All experiments were performed at least thrice and a representative image is shown. Data are expressed as mean ± SD; ^∗∗^*P* < 0.01 versus the control.

Sitagliptin inhibited osteoclastogenesis; thus, we hypothesized that sitagliptin could impair osteoclastic bone-resorptive function. The devitalized bovine bone disks were applied to investigate the influence of 48 h treatment with sitagliptin on mature osteoclast resorptive functions. SEM analysis revealed that osteoclasts actively resorbed the bone surface (**Figure 4A**). The bone resorption activity was effectively inhibited by 12.5 μg/mL sitagliptin and higher concentrations had almost completely blocked the bone resorption activity (**Figures 4A,B**). These data indicated that the administration of sitagliptin effectively inhibited bone resorption *in vitro.*

### Effects of Sitagliptin on F-actin Ring Formation

The formation of an integrated and tight F-actin ring, which is a readily observable characteristic of mature osteoclasts, is a prerequisite for osteoclast bone resorption (Wu et al., 2015; Zhu et al., 2016). Given that sitagliptin inhibits osteoclastic bone resorption, further exploration was performed to examine whether sitagliptin impaired F-actin ring formation. As expected, the characteristic morphology and F-actin ring were observed in the untreated control (Ctrl) osteoclasts as visualized by phalloidin staining and confocal microscopy (**Figure 4C**). However, the number and size of actin ring structures were significantly decreased when the cells were treated with sitagliptin in a dose-dependent manner (**Figure 4C**), thereby indicating that sitagliptin suppressed actin ring formation. In the presence of sitagliptin, F-actin tended to aggregate as small pleomorphic rings that appeared largely unstructured and often varied in size and number (**Figure 4C**).

### Sitagliptin Scavenges ROS Production

**FIGURE 5:**
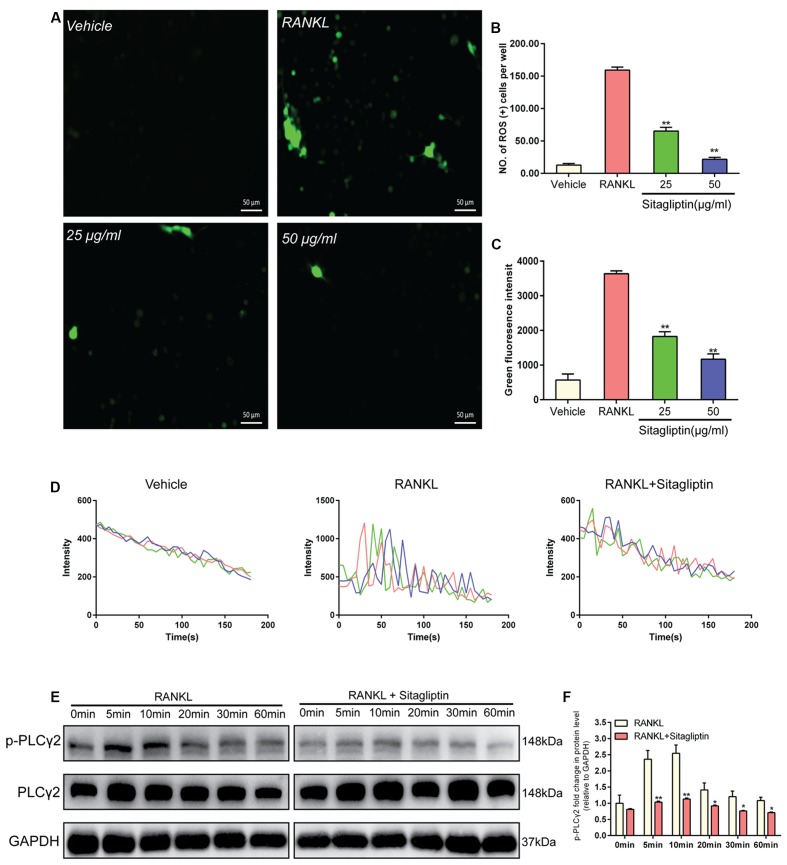
Sitagliptin scavenges for ROS and attenuates Ca^2+^ oscillation. **(A)** Representative images of ROS-positive BMMs during RANKL-induced osteoclastogenesis treated with varying concentrations of sitagliptin (25, 50 μg/mL); scale bars: 50 μm. The ROS positive cell numbers **(B)** and green fluorescence intensity **(C)** in each well (96-well plate) were quantified. **(D)** Representative traces of three randomly selected BMMs in different groups. The fluorescence ratio change was recorded every 5 s for 180 s. **(E)** Western blot analysis of p-PLCγ2 protein expression in BMMs treated with sitagliptin at different time points. GAPDH was used as loading control. **(F)** The band intensities corresponding to p-PLCγ2 were quantified, normalized relative to that of GAPDH, and converted to the fold change of the control. Representative images of three repeated experiments are shown. Data are expressed as mean ± SD; ^∗^*P* < 0.05, ^∗∗^*P* < 0.01 versus those of the RANKL-induced group.

Intracellular ROS was measured with DCFDA, in order to interpret the inhibitory effects of sitagliptin on osteoclastogenesis. BMMs without treatment exhibited low intracellular ROS levels (**Figure 5A**), while BMMs stimulated with RANKL exhibited markedly high intracellular ROS levels, which were supposed to be inhibited by sitagliptin treatments (**Figure 5A**). In addition, both the levels of ROS and the number of ROS-positive cells were obviously decreased by sitagliptin (**Figures 5B,C**).

### Suppression by Sitagliptin of Ca^2+^ Oscillation

Ca^2+^ signaling is essential for the differentiation of osteoclasts; thus, we explored the effects of sitagliptin on Ca^2+^oscillation. When BMMs were treated with M-CSF plus RANKL, Ca^2+^ oscillation was triggered, but not by M-CSF treatment alone. However, sitagliptin inhibits both the frequency of Ca^2+^ oscillations and the average amplitude, which were induced by RANKL (**Figure 5D**). RANKL triggers the activation of PLCγ2 during osteoclast differentiation, resulting in Ca^2+^ mobilization (Negishi-Koga and Takayanagi, 2009). Therefore, we confirmed that sitagliptin could suppress the RANKL-induced Ca^2+^ oscillation through the blockage of RANKL-induced phosphorylation of PLCγ2 (**Figures 5E,F**).

### Sitagliptin Inhibits RANKL-Induced Activation of AKT and ERK Signaling Pathways

**FIGURE 6:**
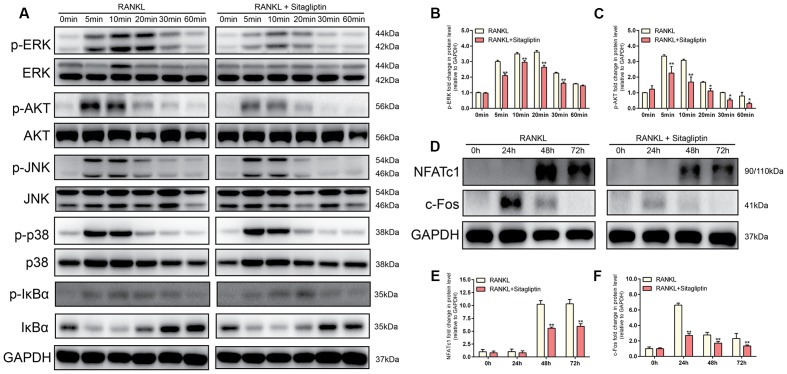
Sitagliptin specifically impaired the RANKL-induced ERK and AKT cascade and suppressed the RANKL-induced activation of NFATc1 and *c-Fos*. **(A,D)** BMMs cells were treated with or without 50 μg/mL sitagliptin, followed by treatment with 50 ng/mL RANKL for the indicated periods. Cell lysates were subjected to Western blot analysis. The band intensities corresponding to p-ERK **(B)**, p-AKT **(C)**, NFATc1 **(E)**, and *c-Fos* **(F)** were quantified, normalized relative to that of GAPDH, and converted to the fold change of the control. Representative images of three repeated experiments are shown. Data are expressed as mean ± SD; ^∗^*P* < 0.05, ^∗∗^*P* < 0.01 versus the RANKL-induced group.

Western blot analysis was performed to investigate the RANKL-induced signaling pathways in order to clarify the underlying mechanism of sitagliptin-induced inhibitory effects of osteoclast formation and function. RAW264.7 cells were stimulated with RANKL (50 ng/mL) for 0, 5, 10, 20, 30, or 60 min to investigate short-term signaling pathways (p-AKT, AKT, p-p38, p38, p-ERK, ERK, JNK, p-JNK, p-IkBa, and IkBa). The data indicated that the phosphorylation of ERK and AKT peaked within 5 min and 10 min, respectively, with RANKL stimulation. However, treatment with sitagliptin significantly suppressed phosphorylation within the aforementioned signaling pathways (**Figures 6A–C**). Collectively, these data suggested that sitagliptin inhibited the ERK and AKT signaling pathways during osteoclastogenesis.

### Suppression by Sitagliptin of *c-Fos* and NFATc1 Expression

**FIGURE 7:**
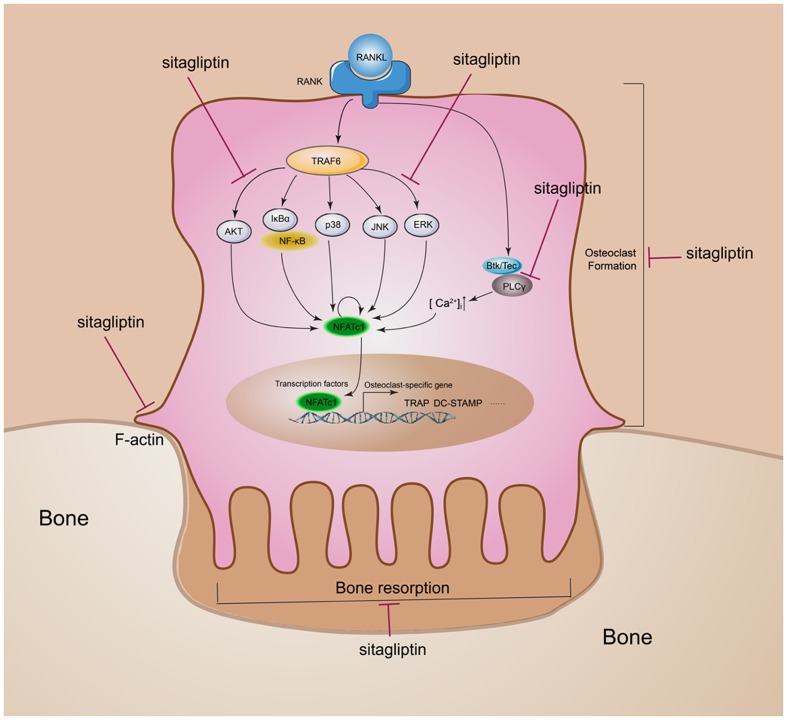
Schematic diagram of the role of sitagliptin in regulating osteoclast differentiation and function. Sitagliptin attenuated osteoclastic bone resorption and F-actin ring formation. Our studies suggest that sitagliptin inhibit osteoclast differentiation through impaired the RANKL-induced ROS, PLCγ2, ERK, and AKT cascade and suppressed the RANKL-induced activation of NFATc1 and *c-Fos*.

The expression of NFATc1, the master transcriptional regulator of osteoclastogenesis, is dependent on *c-Fos* signaling in addition to the MAPK and NF-kB signaling pathways (Nakashima et al., 2012). To identify the long-term signaling pathways (*c-Fos* and NFATc1), BMMs were cultured with or without 50 μg/mL sitagliptin in the presence of M-CSF and RANKL for 0, 24, 48, and 72 h. The results indicated that RANKL treatment of BMMs elevated the *c-Fos* protein expression after 24, 48, and 72 h, whereas downstream transcriptional targets of NFATc1 were sharply induced 48 and 72 h after RANKL treatment (**Figures 6D–F**). However, these effects were strongly suppressed by sitagliptin in a time-dependent manner (**Figures 6D–F**). Taken together, these results indicate that sitagliptin possesses beneficial effects on estrogen deficiency-induced osteoporosis and directly inhibited the differentiation and function of osteoclast (**Figure 7**).

## Discussion

The imbalance of bone homeostasis is a common characteristic of bone lytic diseases. It is the result of the overgrowth of osteoclasts and/or their overactivation and would lead to pathological bone destruction. Osteoclasts, as the major participants, are regarded as key targets for the development of potential anti-osteolytic agents. Recently, some anti-diabetic drugs were demonstrated for anti-osteoclastic bone loss effects, such as metformin (Mai et al., 2011), glyburide (Zinman et al., 2010), exenatide, and liraglutide (Pereira et al., 2015). By contrast, as another type of widely prescribed anti-diabetic drugs, thiazolidinediones clearly demonstrated unfavorable effects on the skeleton, such as activated osteoclastogenesis and impaired osteoblast function according to current preclinical and clinical investigation (Meier et al., 2016). For the first time, this study demonstrated that sitagliptin, which is the first DPP IV inhibitor in anti-diabetic drugs approved by the US FDA, suppresses estrogen deficiency-induced osteoporosis in an OVX mouse model and exerts anti-osteoclastogenic effects by inhibiting the RANKL-induced activation of AKT, ERK, and *c-Fos*, thereby resulting in the inhibition of NFATc1 activity (**Figure 7**).

Estrogen is essential to bone health for its antioxidant activity in osteoclasts and stimulating effects on osteoblasts (Kharkwal et al., 2012; Dou et al., 2016). Postmenopausal osteoporosis is a disease characterized by excessive bone destruction, which is a result of estrogen deficiency. Although the details of these mechanisms are unclear, the increased production of reactive oxygen species (ROS) in osteoclasts and pre-osteoclasts is induced by estrogen deficiency; ROS production is one of the main pathogenic factors in postmenopausal osteoporosis (Lean et al., 2003; Grassi et al., 2007; Bartell et al., 2014). ROS plays an essential role in RANKL-induced osteoclastogenesis. RANKL and M-CSF elevate ROS levels in osteoclast progenitors and the generated ROS act as an intracellular signal mediator to activate MAPKs in osteoclast differentiation, activation, and survival (Bartell et al., 2014). The antioxidant potential of sitagliptin was depicted in a transient cerebral ischemia/reperfusion injury animal model, where it reduced neutrophil infiltration, as well as the lipid peroxides and nitric oxide associated with the replenishment of reduced glutathione (El-Sahar et al., 2015). When stimulated by RANKL in this study, the pre-osteoclasts exhibited obviously high intracellular ROS levels, which were inhibited by sitagliptin treatments in a dose-dependent manner (**Figures 5A,B**). Therefore, the results indicated that sitagliptin induced the inhibition of osteoclastogenesis, including the ROS scavenging activity.

RANKL and M-CSF play critical roles in osteoclastogenesis (Boyle et al., 2003). The binding of RANKL to the RANK receptor triggers the activation of downstream signaling molecules, such as MAPKs, AKT, and PLCγ, to subsequently induce the activation of transcription factors, such as *c-Fos* and NFATc1, thereby regulating the expression of genes required for osteoclast differentiation (Yeon et al., 2014). In the present study, sitagliptin suppressed the RANKL-induced phosphorylation of ERK without affecting p38 and JNK. ERK phosphorylation is the main regulator of *c-fos* expression in osteoclast precursors (Okawa et al., 2009). When ERK signaling is still active, *c-Fos* is phosphorylated by sustained ERK. However, when ERK activation is transient, its activity is attenuated before the *c-Fos* protein accumulates; under these conditions, c-Fos is unstable (Murphy et al., 2002; Okawa et al., 2009). Consistent with the previous studies, our data indicated that ERK inactivation mediated by sitagliptin is associated with *c-fos* downregulation (**Figures 6A,D**). Moreover, sitagliptin also attenuated the RANKL-induced phosphorylation of AKT. AKT is very important in osteoclasts survival instead of osteoclasts differentiation (Gingery et al., 2003). However, recent studies showed the importance of the AKT–NFATc1 signaling axis in osteoclast differentiation (Moon et al., 2012; Baek et al., 2016). Aside from regulating NFATc1 expression during osteoclastogenesis, cells with *Akt* overexpression significantly induced NFATc1 translocation to the nucleus rather than the cytoplasmic region (Baek et al., 2016). AKT is a well-known downstream effector of PI3K. It was reported that the inhibition of PI3K or AKT disrupted the F-actin ring formation of osteoclasts (Matsumoto et al., 2011). Sitagliptin inhibited the RANKL-induced phosphorylation of AKT (**Figure 6A**) and impaired F-actin ring formation (**Figure 4C**). Therefore, sitagliptin may negatively regulate the translocation of NFATc1 by modulating the AKT signaling pathway, subsequently leading to cytoskeletal organization of osteoclasts.

In the previous study, RANKL also triggered the phosphorylation of PLCγ during osteoclast differentiation, subsequently leading to Ca^2+^ mobilization (Negishi-Koga and Takayanagi, 2009). By phosphorylating the tyrosine residues, the PLCγ family members PLCγ1 and PLCγ2 exert their catalytic activity to regulate protein kinase C activation and intracellular Ca^2+^ levels in hematopoietic cells (Wilde and Watson, 2001). However, the phosphorylation of PLCγ2, but not PLCγ1, is required for RANKL-mediated Ca^2+^ signaling in osteoclast differentiation (Mao et al., 2006). The RANKL-mediated Ca^2+^ mobilization and influx provides the Ca^2+^ signaling, which induces osteoclasts to upregulate and autoamplify NFATc1, which is essential for osteoclast differentiation, cytoskeleton organization, and bone resorption activity (Hwang and Putney, 2011; Kajiya, 2012). Our data clearly confirmed that sitagliptin reduced Ca^2+^ oscillation (**Figure 5D**) and blocked the phosphorylation of PLCγ2 (**Figures 5E,F**). The findings of the present study suggest that the anti-osteoclastogenic activity of sitagliptin involves the inhibition of PLCγ-dependent Ca^2+^ oscillation.

Our study indicated that sitagliptin exerted anti-resorptive effects by weakening the ability of mature osteoclasts in bone resorption (**Figure 4A**). A complex of a high density of functional V-ATPase and well-polarized F-actin ring was formed and clustered on the ruffled border membrane of osteoclasts as prerequisites for efficient bone resorption (Boyle et al., 2003; Zaidi et al., 2003). We did not investigate the effect of sitagliptin on the V-ATPase complexes of osteoclasts. However, sitagliptin effectively inhibited F-actin ring formation in osteoclasts (**Figure 4C**). The anti-resorptive effect of sitagliptin on mature osteoclasts was confirmed to be associated with the quick disruption of the actin ring structure, which subsequently leads to the generalized changes to cytoskeletal integrity. As sitagliptin can effectively suppress NFATc1 activity, the disruption of cytoskeletal integrity and F-actin ring formation could be associated with the inhibition of integrin αvβ3 and c-Src tyrosine kinase activities, which are both late gene targets of NFATc1 (Zhu et al., 2016).

As expected, the administration of sitagliptin protected against estrogen deficiency-induced bone loss *in vivo*, which is consistent with its anti-osteoclastogenesis and anti-resorption effects *in vitro.* In addition, the current results indicated that sitagliptin greatly reduced the number of TRAP^+^ osteoclasts and the bone resorption induced by OVX. However, bone homeostasis depends on a functional balance between bone-resorbing osteoclasts and bone-forming osteoblasts. Further investigations are needed to understand the signaling mechanisms that mediate the effects of sitagliptin on osteoblasts. Then we found that concentrations of up to 50 μg/mL sitagliptin had no cytotoxic effects on BMMs, which were treated with sitagliptin for 24 h and 72 h (**Supplementary Figure S1A**) and sitagliptin had no effects on the osteogenic differentiation of BMSCs detected by alkaline phosphatase (ALP) and Alizarin red S staining (**Supplementary Figures S1B,C**).

Taken together, our results demonstrated the inhibitory effects of sitagliptin on osteoclast function and osteoclastogenesis *in vivo* and *in vitro*. This study also identified that sitagliptin functioned by suppressing the AKT and ERK signaling pathways, scavenging ROS activity, and suppressing the Ca^2+^ oscillation, and consequently the expression and/or activity of the osteoclast-specific transcription factors NFATc1 and *c-Fos* affects were affected. Further studies should focus on identifying the target binding molecules of sitagliptin, the mechanism by which sitagliptin inhibits the fusion of preosteoclasts, and the pit formation of mature osteoclasts.

## Author Contributions

CW, FX, XC, and XZ: conception and design; CW, FX, XQ, GH, ZZ: experiments and/or data analysis; XC: clinical consultancy; XZ: intellectual input and supervision; CW and FX: article writing with contributions from other authors.

## Conflict of Interest Statement

The authors declare that the research was conducted in the absence of any commercial or financial relationships that could be construed as a potential conflict of interest.

## Supplementary Material

FIGURE S1 The effect of sitagliptin on the osteogenic differentiation of BMSCs. **(A)** CCK-8 was performed in triplicate to analyze the cell viability of BMSCs treated with varying doses of sitagliptin for 24 and 72 h. **(B)** BMSCs were incubated with osteogenic differentiation medium plus indicated concentrations of sitagliptin. Alkaline phosphatase (ALP) staining measured after 7 days osteogenic induction. **(C)** BMSCs were incubated with osteogenic differentiation medium plus indicated concentrations of sitagliptin. With 14 days osteogenic induction, the differentiated BMSCs were stained with Alizarin red S to detect bone nodules. Representative images of three repeated experiments are shown.

## Abbreviations

BMD: bone mineral density
BV/TV: trabecular bone volume per tissue volume
Calcr: calcitonin receptor
DAPI: 4′ 6-diamidino-2-phenylindole
Dc-stamp: dendrite cell-specific transmembrane protein
DPP IV: dipeptidyl peptidase IV
ERK: extracellular signal-regulated kinase
JNK: c-jun N-terminal kinase
M-CSF: macrophage colony-stimulating factor
Nfatc1: nuclear factor of activated T-cells cytoplasmic 1
OVX: ovariectomized
PBS: phosphate-buffered saline
PLCγ: phospholipase Cγ
RANKL: receptor activator of nuclear factor-κB ligand
SMI: structure model index
Tb.N: trabecular number
Tb.Sp: trabecular separation
TRAP: tartrate-resistant acid phosphatase
Tb.Th: trabecular thickness.
