# Supplementary figures and images for: Sitagliptin, An Anti-diabetic Drug, Suppresses Estrogen Deficiency-Induced OsteoporosisIn Vivo and Inhibits RANKL-Induced Osteoclast Formation and Bone Resorption In Vitro
Source: Front Pharmacol. 2017 Jun 30;8:407. doi: 10.3389/fphar.2017.00407 (PMC5492451; doi:10.3389/fphar.2017.00407)

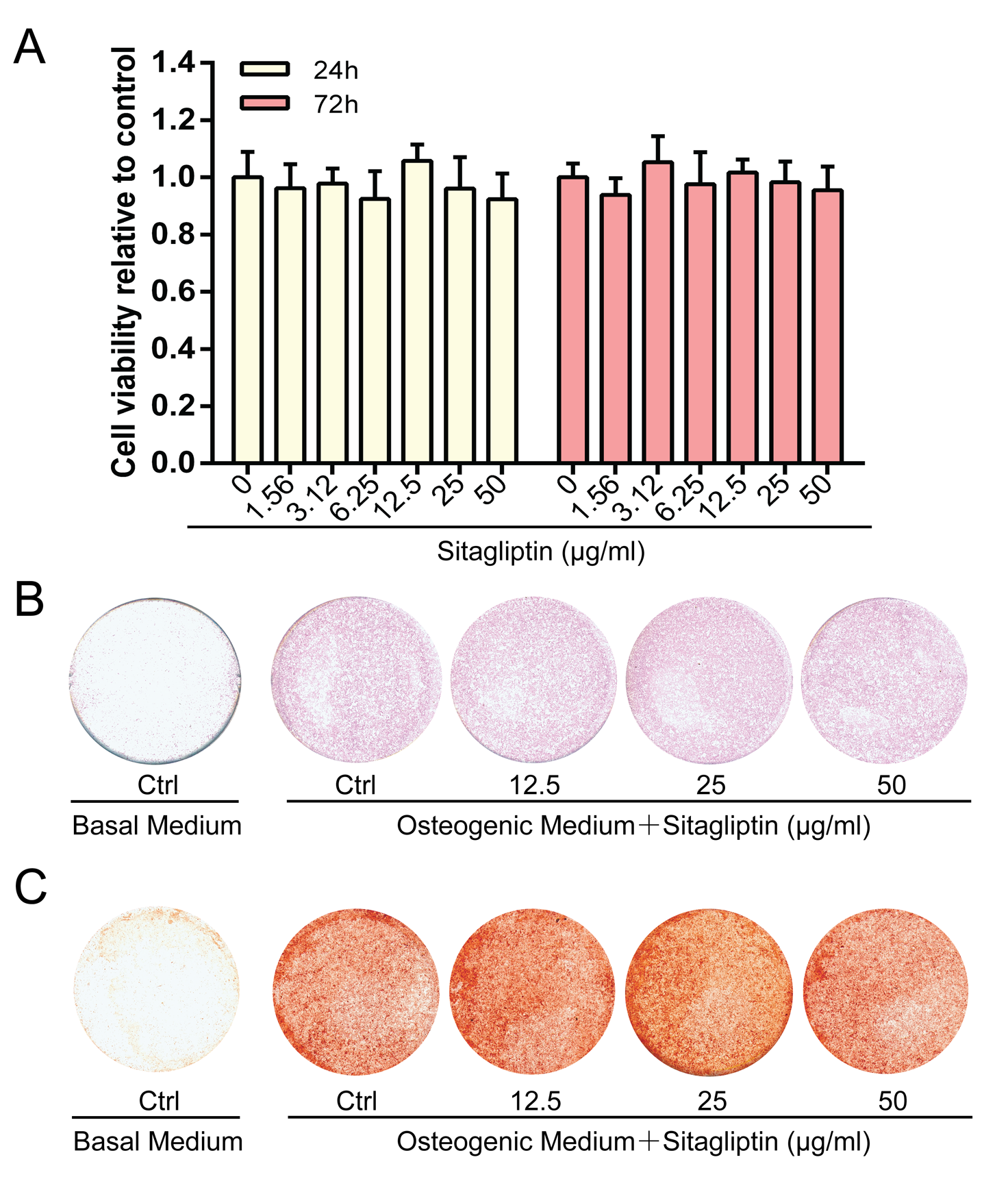

Supplement: FIGURE S1 — The effect of sitagliptin on the osteogenic differentiation of BMSCs. (A) CCK-8 was performed in triplicate to analyze the cell viability of BMSCs treated with varying doses of sitagliptin for 24 and 72 h. (B) BMSCs were incubated with osteogenic differentiation medium plus indicated concentrations of sitagliptin. Alkaline phosphatase (ALP) staining measured after 7 days osteogenic induction. (C) BMSCs were incubated with osteogenic differentiation medium plus indicated concentrations of sitagliptin. With 14 days osteogenic induction, the differentiated BMSCs were stained with Alizarin red S to detect bone nodules. Representative images of three repeated experiments are shown. [file Image_1.TIF]
